# Grafting enhances plants drought resistance: Current understanding, mechanisms, and future perspectives

**DOI:** 10.3389/fpls.2022.1015317

**Published:** 2022-10-06

**Authors:** Le Yang, Linchao Xia, Yi Zeng, Qingquan Han, Sheng Zhang

**Affiliations:** ^1^ Key Laboratory of Bio-Resource and Eco-Environment of Ministry of Education, College of Life Sciences, Sichuan University, Chengdu, China; ^2^ The Engineering Research Institute of Agriculture and Forestry, Ludong University, Yantai, China

**Keywords:** scion, rootstock, drought tolerance, phytohormones, miRNA

## Abstract

Drought, one of the most severe and complex abiotic stresses, is increasingly occurring due to global climate change and adversely affects plant growth and yield. Grafting is a proven and effective tool to enhance plant drought resistance ability by regulating their physiological and molecular processes. In this review, we have summarized the current understanding, mechanisms, and perspectives of the drought stress resistance of grafted plants. Plants resist drought through adaptive changes in their root, stem, and leaf morphology and structure, stomatal closure modulation to reduce transpiration, activating osmoregulation, enhancing antioxidant systems, and regulating phytohormones and gene expression changes. Additionally, the mRNAs, miRNAs and peptides crossing the grafted healing sites also confer drought resistance. However, the interaction between phytohormones, establishment of the scion-rootstock communication through genetic materials to enhance drought resistance is becoming a hot research topic. Therefore, our review provides not only physiological evidences for selecting drought-resistant rootstocks or scions, but also a clear understanding of the potential molecular effects to enhance drought resistance using grafted plants.

## Introduction

Water is essential for the growth and development of plants, and it accounts for 70%-90% of the plant’s fresh weight. With global climate change, arid and semi-arid areas are accounting for ~40% of global land area and will cross over 50% by the end of the 21st century (Huang et al., 2015). This will cause a severe water shortage for plants in this area, resulting in a series of morphological, physiological, and molecular changes in them (Ahmed et al., 2009; Liu et al., 2014; Kumar et al., 2017). For example, drought reduces the plant cell’s water potential, transpiration rate, turgor pressure, and photosynthetic rate, while increasing their reactive oxygen species (ROS) accumulation, which causes poor plant growth, early flowering, and ultimately plant yield losses (Alan et al., 2007; Kumar et al., 2017; Chaimala et al., 2020; Gupta et al., 2020; Zia et al., 2021). According to the past decade’s data, drought caused global crop yield losses of ~$30 billion (Gupta et al., 2020). With the rapid growth of global population and moderate increase in global arable land, water demand for crop growth could double by 2050, whereas the available freshwater is predicted to drop by 50% due to climate change (Gupta et al., 2020). Under such conditions, as plants are stationary, it is crucial to cultivate plants that can adapt to the arid environment and maintain their normal yield and growth. Additionally, using artificial methods (*e.g.*, drought hardening, directive breeding, and exogenous application of plant growth regulators) can also improve plant drought resistance (Farooq et al., 2009; Tesfahun and Yildiz, 2018). Interestingly, grafting has shown promise in improving plant drought resistance through the use of drought-resistant plant materials.

Grafting is a type of asexual propagation method that connects the budding stem segment of one plant (scion) to that of another plant containing roots (rootstock), to allow them to grow together. It is widely used in commercial fruit and vegetable cultivation, landscaping, and for verifying molecular movement in plants (Fullana-Pericàs et al., 2018; Li et al., 2020). Moreover, it can promote the growth and development of the grafted plants, maintain the excellent properties of the parents, change their branch structure, increase plant yield, positively influence fruit flavor, improve nutritional value, and enhance abiotic stress resistance (Sánchez-Rodríguez et al., 2014; Melnyk and Meyerowitz, 2015; Thomas and Frank, 2019; Ellenberger et al., 2021). Unfortunately, grafting cannot be applied to all plants, as it is determined by grafting compatibility (Habibi et al., 2022). Grafting compatibility means the successful connection between the vascular and non-vascular systems at the grafted junction. Generally, the survival rate of intra-generic grafting is high, while that of inter-genera is low. Recently, studies on the physiological and molecular mechanisms of grafting junction have gradually become a new hotspot, like the key responsive genes in grafted healing sites (Melnyk et al., 2018; Notaguchi et al., 2020; Kurotani and Notaguchi, 2021; Thomas et al., 2021; Chambaud et al., 2022) and genetic information exchange between rootstocks and scions (Thomas and Frank, 2019; Yang et al., 2019; Cerruti et al., 2021; Okamoto et al., 2022).

Since drought greatly influences plant growth and development, it is essential to learn how to mitigate the negative effects through grafting (Zia et al., 2021). Grafting improves plant drought resistance (Ellialtio­glu et al., 2019; Lopez-Serrano et al., 2019; Chen et al., 2020), which is primarily determined by the rootstock, despite the scion also affecting the grafted plant (Han et al., 2013; Han et al., 2019; Chen et al., 2020; Lopez-Hinojosa et al., 2021). Despite there being multiple studies and reviews on grafting and drought resistance, few have summarized how the molecular response of grafted plants is linked to their physiology and phenotype under drought stress. Therefore, this paper mainly summarizes the research to date on molecular mechanisms and physiological and morphological changes in grafted plants, while also listing the drought responsive genes and mobile molecules that generate drought resistance in grafted plants (**Tables 1**, **2**).

**Table 1:** Grafting influences the expression of drought responsive genes.

Category	Gene name	Effects	Scion/Rootstock	Reference
Substance transport	*SWEET 14*	Up-regulated, transporting sugars, promoting the accumulation of carbohydrates in roots, and providing energy for root elongation and growth	(*Vitrus. vinifera*/(*V. rupestris × V. berlandieri*)	Yildirim et al., 2018
	*NRT1/PTR*	Up-regulated, transporting proteins, promoting nitrogen accumulated in roots and providing energy for root elongation and growth		
Osmoregulation	*P5SC*	Up-regulated, regulating proline biosynthesis and alleviating osmotic stress	(*P. persica*/(*P. dulcis × P. persica*)	Jimenez et al., 2013
	*SIP1*	Up-regulated, regulating raffinose biosynthesis and alleviating osmotic stress		
	*LEA5*, *ERD10C*	Up-regulated, preventing cell dehydration	*Nicotiana tabacum/N. tabacum*	Liu et al., 2014
	*BFRUCT3*, *SPSIF*, *SUS3 and 4*	Up-regulated, regulating glucose and sucrose biosynthesis, stabilizing cellular membranes and maintaining cell turgor	*Citrus sinensis* (L.) Osbeck*/C. limonia* Osbeck	Goncalves et al., 2019
Water reservation	*TIP1*, *TIP2* and *PIP*	Down-regulated in severe drought conditions, preventing water escaping from roots into soil and maintaining water in roots	*V. vinifera/*(*V. rupestris* × *V. berlandieri)*	Yildirim et al., 2018
ABA biosynthesis	*NCED1*	Up-regulated, involving in ABA biosynthesis and increasing ABA content	*C. sinensis/C. limonia*	Allario et al., 2013
	*ABCG22*		*Cucumis sativus/Luffa cylindrica*	Liu et al., 2016
ABA-dependent signaling pathway	*YTP1*	Up-regulated, induced *RD22* and *ABF*, involving in ABA-dependent signaling pathway	Overexpression *MhYTP1 Malus domestica*/*M. domestica*	Guo et al., 2019
	*RD22*	Up-regulated, participating in ABA signal transduction process		
	*AREB*		*N. tabacum*/*N. tabacum*	Liu et al., 2014
	*PYR/PYL*		*Lycopersicum esculentum '112'/L. esculentum '606'*	Zhang et al., 2019b
	*SnRK2*			
	*AREB/ABF*			
	*PP2C*		*V. vinifera/*(*V. vinifera* ×*V. berlandieri*) × *V. berlandieri*	Prinsi et al., 2021
ABA-independent signaling pathway	*DREB2A*	Up-regulated, stimulating drought responsive genes	*C. sinensis* (L.) Osbeck*/C. limonia* Osbeck	Goncalves et al., 2019
Antioxidative system	*NRX*	Up-regulated, protecting the ROS scavenging capacity of CAT	*C. sativus/Cucurbita moschata*	Davoudi et al., 2022
	*CAT*	Up-regulated, clearing the ROS		
	*SOD*, *APX*		*C. sinensis* (L.) Osbeck*/C. limonia* Osbeck	Goncalves et al., 2019
	*CHY1*	Up-regulated, involving in carotenoid biosynthesis		
Photosynthesis	*RbcL* and *RbcS*	Up-regulated, maintaining Rubisco expression and activity and improving photosynthetic performance	*Chrysanthemum morifolium/Artemisia annua*	Chen et al., 2018
	*Cab*	Up-regulated, encoding chlorophyll a binding protein and maintaining chlorophyll and photon absorption		
	*psaB*	Up-regulated, involving in enhancing PS I activity		
Casparian band	*COMT*	Up-regulated, contributing to suberin and lignin biosynthesis	(*V. vinifera*/(*V. rupestris × V. berlandieri*)	Yildirim et al., 2018
	*eceriferum 3*	Up-regulated, regulating wax biosynthetic processes		
	*KCS*	Up-regulated, connected with the suberization		

Genes that have similar function are gathered together in the first row named ‘category’. The names and functions of each differentially expressed genes in the grafted plants, the grafted combinations that were found those genes and the cited references are listed in the other rows of table.

**Table 2:** The exchanging molecules between scions and rootstocks to improve drought resistance.

Category	Mobile molecules	Effects	Scion/Rootstock	Reference
Antioxidants	HSP70	Alleviating lipid peroxidation and protecting cell membrane integrity	*Cucumis sativus/Cucurbita moschata*	Davoudi et al., 2022
	HSP81			
	*Prx*	Improving peroxidase activity		
	DnaJ	Maintaining the stability of intracellular protein components and is related to protein domain specific binding		
	DnaJ-like B8	Maintaining the stability of intracellular protein components and is related to protein folding		
Photosynthesis	*psbB*	Binding chlorophyll and helping catalyze the primary light-induced photochemical processes of PSII		
	*psbD*	Assembling stable PSII complexes		
	*LhcI*	Promoting light energy collection and energy transfer to photosynthetic reaction centers		
	*psaA*	Encoding P700 chlorophyll A1 apolipoprotein and involving in PSI response process		
	*psaB*	Encoding P700 chlorophyll A2 apolipoprotein and involving in PSI response process		
ABA biosynthesis and response	STK	Maintaining the integrity of cell membrane and is related to protein phosphorylation		
	*DRM*	Improving drought resistance and is related to ABA response progress	*Pyrus bretchneideri/P. betulaefolia*	Hao et al., 2020
	*NDUFB7*	Related to ABA signaling, stomatal aperture and improving drought resistance	*Nicotiana benthamiana/Arabidopsis thaliana*	Notaguchi et al., 2015
	CLE25	Inducing *NCED3* expression and ABA accumlation	cle25 #10 *A. thaliana/*wild-type *A. thaliana*	Takahashi et al., 2018
Root regulation	miR160	Regulating RSA adjustment	Mdm-miR160e OE *Malus domestica*/GL-3 *M. domestica*	Shen et al., 2022

The category is composed of the molecules that has the same functions. The molecules in table are mainly miRNAs. Only the CLE25 is a kind of peptides and the miR160 is a kind of miRNAs. The last two rows demonstrate which the grafted combinations and references the molecules are from.

## Rootstock changes

**Figure 1:**
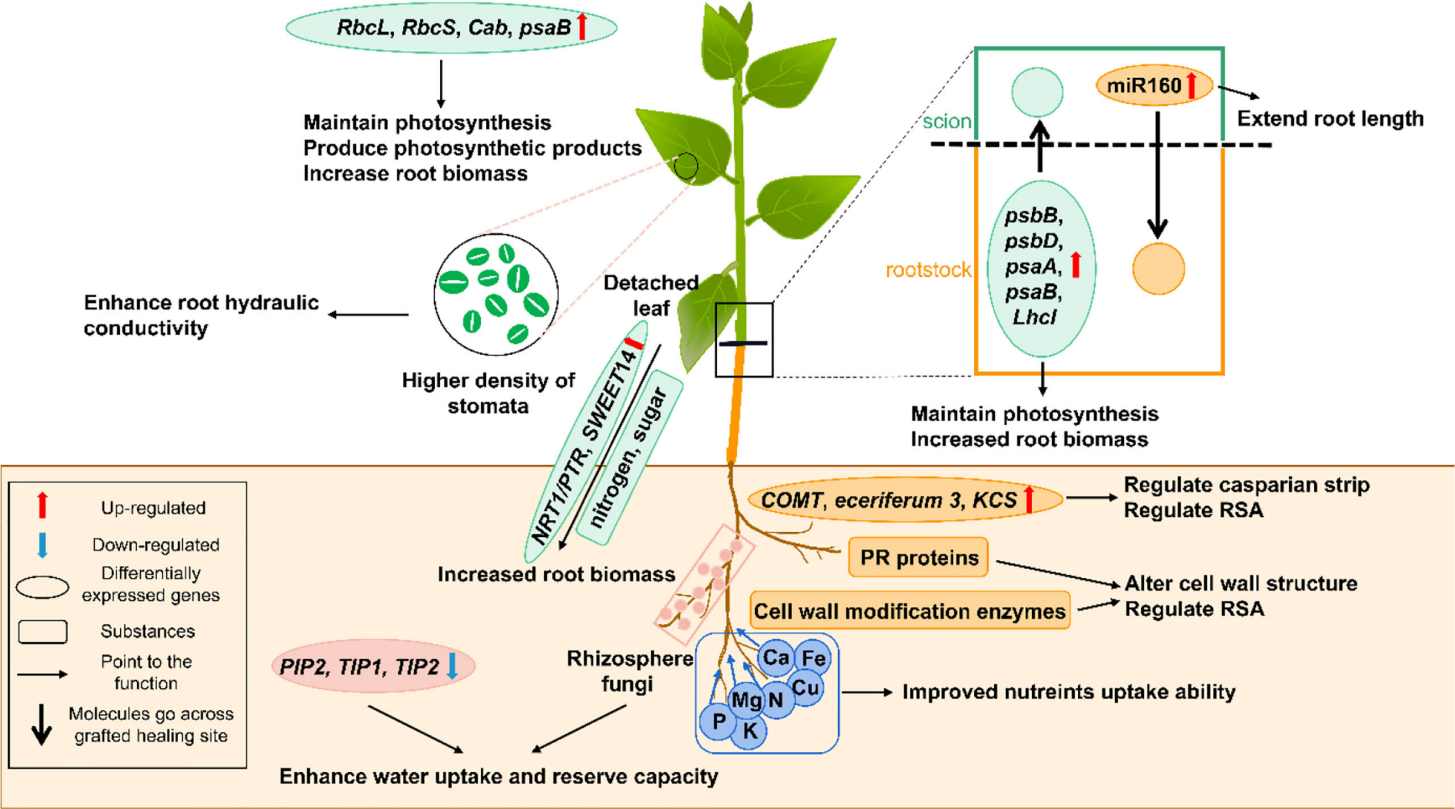
A schematic model shows the physiological and molecular mechanisms that enhancing drought resistance by root and shoot regulation in grafted plants. The green part represents for the scion, and the brown part represents for the rootstock. The black box shows the figure notes. *Cab*, *chlorophyll a/b binding protein*; *COMT*, *caffeic acid 3-O-methyltransferase*; *KCS*, *3-ketoacyl-CoA synthase*; *LhcI*, *photosystem I light-harvesting chlorophyll a/b-binding protein*; *NRT1/PTR*, *nitrate transporter 1/peptide transporter*; *PIP*, *plasma membrane intrinsic proteins*; *psaA*, *photosystem I P700 chlorophyll a apoprotein A1*; *psaB*, *photosystem I P700 chlorophyll a apoprotein A2*; *psbB*, *photosystem II CP47 reaction center protein*; *psbD*, *photosystem II protein D2*; *RbcS*, *ribulose-1,5-bisphosphate carboxylase/oxygenase small subunit*; *RbcL*, *Ribulose-1,5-diphosphate carboxylase/oxygenase large subunit*; *SWEET14*, *bidirectional sugar transporter*; *TIP1* and *2, tonoplast intrinsic proteins 1* and *2*.

The root is an important organ for water and nutrient uptake from the soil to support plant growth and development. Drought usually reduces root biomass, damages root system architecture (RSA) and decreases root hydraulic conductivity (Yang et al., 2021; Zia et al., 2021). As shown in **Figure 1**, drought-resistant grafted plants usually mitigate these effects by appropriately adjusting their roots.

### Morphological changes of rootstock

Increased root biomass and root-shoot ratio confer great advantages to grafted plants under drought stress (Li et al., 2020). The grafted grapevine and pepper plants display enhanced drought resistance by maintaining high root biomass and water absorption and storage ability (Lopez-Serrano et al., 2019; Prinsi et al., 2021). The accumulation of carbohydrates and nitrogen which need transporter proteins to move to drought-resistant roots, on the one hand, is from detached leaves, and on the other hand, is from photosynthesis (Yildirim et al., 2018; Han et al., 2019). The bidirectional sugar transporter (SWEET14) and nitrate transporter 1/peptide transporter (NRT1/PTR), were two significantly accumulating proteins in drought-resistant grapevine rootstock 110R, which promoted the carbohydrates and nitrogen accumulation in roots (Chen et al., 2012; Yildirim et al., 2018; Ji et al., 2022). Although we believe that during drought stress, transporter proteins will accumulate in the grafted plants, the underlying regulated pathways need further investigation.

Besides accumulating greater root biomass, drought-resistant rootstocks display higher root plasticity and root vigor than drought-sensitive ones (Han et al., 2019; Silva et al., 2021). Maintaining the rapid growth and large roots under water stress is important for drought-resistant improvement (Li et al., 2020). By using the accumulated assimilate, the root system architecture (RSA) (*e.g.*, root length, root diameter, root area, and root volume) of drought-resistant grapevine rootstocks changes to better absorb the water and nutrients in deep soil, with the root hydraulic conductivity being greater than drought-sensitive ones (Alsina et al., 2011; Sucu et al., 2017; Yildirim et al., 2018). Similar results were obtained in tomatoes (Suchoff et al., 2018). These root architecture changes depend on signal molecules and multiple genes. Glutamate is a common signaling molecule in RSA under drought stress (Qiu et al., 2019). Decreased glutamate in drought-resistant citrus rootstocks during severe drought stress inhibited the growth of lateral roots, which allocated more resources towards the primary root elongation to expand their reach of the available water supply (Sousa et al., 2022). Additionally, the up-regulation of genes encoding cell wall modification enzymes and pathogenesis-related (PR) proteins in the rootstock can also alter cell wall structure to easily modify the RSA to enhance the water uptake capacity (Sels et al., 2008; Yildirim et al., 2018). The miR160 moving from scion to rootstock in the grafted apple and tobacco plants improved root development by extending the root length (Shen et al., 2022).

### Root water preservation and nutrient uptake

Root biomass accumulation and RSA regulation are the two primary water storage methods in roots. Besides these, genes related to suberization and wax biosynthesis, like *caffeic acid 3-O-methyltransferase* (*COMT*), *eceriferum 3* and *3-ketoacyl-CoA synthase* (*KCS*), were up-regulated to build and thicken the Casparian strip (Vincent et al., 2005; Lee et al., 2009; Li et al., 2015; Yildirim et al., 2018). The Casparian strip helped prevent the water backflow from the root to the soil (Yildirim et al., 2018). Aquaporins, are water channels encoded by *plasma membrane intrinsic proteins* (*PIPs*) and *tonoplast intrinsic proteins* (*TIPs*) genes, and their expression pattern varied with the extent of drought stress and plant species (Gautam and Pandey, 2021; Shivaraj et al., 2021). The *PIP* genes were differentially expressed in the grafted plants of two plum rootstocks (R20 and R40), but it was difficult to explain their drought-induced expression patterns and their effects (Opazo et al., 2020). However, during severe drought-stressed conditions, *TIP1*, *TIP2*, and *PIP2* were down-regulated in the grapevine rootstocks, which reduced aquaporins and prevented water loss from roots (Afzal et al., 2016; Yildirim et al., 2018). The *PIP1;2* was considered a candidate gene for improving the plant water conductivity in grafted hickory plants (Kumar et al., 2018). Additionally, aquaporins were reported to be transported from pumpkin rootstocks to cucumber scions during drought (Davoudi et al., 2022). Therefore, aquaporins and their related genes need further exploration in grafted plants under drought stress. Furthermore, the root’s water and nutrient uptake ability was improved in the grafted poplars, which may be related to the high relative abundance of rhizosphere fungi (Liu et al., 2019).

The plant nutrient uptake and utilization depend on the root’s nutrient absorption capacity or the strength of signals arising from the scions (Savvas et al., 2010). Obviously, grafting with vigorous drought-resistant rootstocks can enhance the nutrient uptake ability to improve plant yield when compared to self-rooted plants (Savvas et al., 2010; Zhang et al., 2020). For example, compared with the scions grafted with drought-sensitive tomato rootstocks ‘S’, the concentrations of vital macronutrients like N, P, K, Ca, and Mg were higher in the leaves of scions grafted with drought-resistant ‘T’ under drought-stressed conditions, thereby indicating that drought-resistant rootstocks enhanced the uptake and translocation of nutrients toward the shoots (Zhang et al., 2020). Similarly, plants grafted with drought-tolerant tomato rootstocks (Zarina) showed higher macronutrient (N, P and K) and micronutrient (Fe and Cu) concentrations under water stressed conditions (Sánchez-Rodríguez et al., 2013; Sánchez-Rodríguez et al., 2014). Water stress may also inhibit the nitrogen metabolism enzymes, thereby limiting the plant’s nitrogen assimilation ability (Sánchez-Rodríguez et al., 2013). When drought-tolerant tomato variety was used as rootstocks, the grafted plants showed an improved N uptake and NO^3-^ assimilation, which promoted their growth (Sánchez-Rodríguez et al., 2013).

## Scion changes

Scions maintain their own growth and development through photosynthetic products. The drought stress-induced adverse effects on scions included leaf wilting, reduction of leaf area and numbers, reduced leaf and stem biomass, and weakened photosynthesis, all of which decreased the photosynthetic products and water contents, and water use efficiency of scions (Shao et al., 2008). Grafting onto drought-resistant rootstocks can be a good strategy to alleviate these problems (Shehata et al., 2022).

### Morphological changes of the scion

Morphological observations post water deficit conditions showed a lower proportion of yellow and dry leaves in the scions grafted onto drought-resistant grapevine rootstocks (Sucu et al., 2017). The leaf area size directly affects plant photosynthetic intensity (Yang et al., 2021). Under the drought treatment, the drought-resistant apple rootstocks had a greater leaf area, which improved photosynthesis, thereby positively affecting the whole plant productivity (Valverdi and Kalcsits, 2021). Shoot growth is sensitive to water stress and may stop even with minor water reduction (Sabir and Kucukbasmaci, 2019). Naturally, the drought-resistant grapevine rootstocks were found to maintain the shoot growth of scions under water deficit conditions. (Sabir and Kucukbasmaci, 2019). Fresh and dry weights of the leaf and shoot are considered important indexes to screen and identify drought-tolerant genotypes (Bikdeloo et al., 2021). When scions are grafted onto drought-tolerant grapevine rootstocks, their leaf dry weight were higher than the grafted plants containing sensitive rootstocks (Gullo et al., 2018). Plants grafted onto high vigor watermelon rootstocks exhibited a relatively lower reduction in growth and shoot biomass (Ali et al., 2019; Bikdeloo et al., 2021).

The micro-morphological changes of leaves and stems also promoted the water absorption of scions. Drought-resistant rootstocks increased the diameter and density of the xylem vessels and decreased the numbers of emboli at the grafted site to increase the hydraulic conductance capacity and ultimately altered the drought resistance of the scion (Bauerle et al., 2011). When two plum scions (An and Np) were grafted onto the same rootstocks, the grafted combination with An scion had higher root hydraulic conductivity, probably due to the higher stomatal density of scions, thereby causing great internal pressure in the transpiration stream (Opazo et al., 2020). Using scions and rootstocks of xeric origin can not only improve the drought tolerance of grafted plants, but also shape their phenology, including delaying bud-break and reducing stem secondary growth of trees (Camisón et al., 2021).

### Photosynthesis

Scions that are grafted with drought-resistant tomato rootstocks demonstrated a lower photosynthetic rate reduction (Alves et al., 2021). The maintenance of a high CO_2_ assimilation rate in drought-resistant grafted plants provides the basis for good plant growth and productivity under long-term drought situations. This causes drought-resistant grafted plants to accumulate greater biomass accumulation than their drought-sensitive counterparts, despite this accumulation being lesser than during normal conditions. Photosynthesis is adversely affected by stomatal and non-stomatal factors. The stomatal movement and development will be discussed later. The non-stomatal factors are the main photosynthesis inhibitory factors and they include chloroplast rupturing, inhibition of chlorophyll synthesis, a decrease of photosystem II reaction center activity, and inhibition of ribulose-1,5-diphosphate carboxylase/oxygenase (Rubisco) activity (Flexas and Medrano, 2002; Flexas et al., 2004; Twalla et al., 2021). As shown in **Figure 1**, these problems are alleviated by grafting onto high vigor rootstocks. The chlorophyll content is a useful index for evaluating plant drought resistance. The chlorophyll content of leaves was the highest in the scions grafted with the strongest drought-resistant rootstocks among three different rootstocks during drought stress treatment, which also indicated higher photosystem II activity (Dong et al., 2021). The *Rubisco large subunit* (*RbcL*), *Rubisco small subunit* (*RbcS*), *chlorophyll a/b binding protein* (*Cab*) and *photosystem I p700 chlorophyll a apoprotein A2* (*psaB*) genes were up-regulated in chrysanthemum scions of the drought-resistant grafted plants and it helped maintain the photosynthetic performance (Chen et al., 2018; Wilson and Hayer-Hartl, 2018). The movement of a series of mobile mRNAs from the pumpkin roots to the scions, also called scion-rootstock communication, can also improve the photosynthetic performance during drought conditions (Davoudi et al., 2022). Specifically, multiple genes, including *photosystem II CP47 reaction center protein* (*psbB*), *photosystem I p700 chlorophyll a apoprotein A2* (*psaB*), *photosystem II protein D2* (*psbD*), *photosystem I p700 chlorophyll a apoprotein A1* (*psaA*) and *photosystem I light-harvesting chlorophyll a/b-binding protein* (*LhcI*), functional in different photosynthesis-related processes, were induced to maintain the photosynthetic activity of the grafted plants (Davoudi et al., 2022). Among them, *psaB* is the common gene that was not only detected in chrysanthemum scions, but also identified in the molecule movement from pumpkin rootstocks to cucumber scions. Therefore, we believe that some photosynthesis-related genes are being activated in the roots and their transcripts are being moved to scions.

## Crop yield and quality

For trees, improving drought resistance through grafting is the most important aspect, as it allows better growth under drought stressed conditions. However, for grafted vegetables and fruits, it is not only important to improve their drought resistance, but also to retain adequate yield and high vegetable/fruit quality in the process (Fullana-Pericàs et al., 2020). Many studies have suggested that the drought-induced negative effects on the yield and quality of fruits and vegetables can be mitigated by grafting with drought-resistant rootstocks (**Supplementary Table 1**) (Ellenberger et al., 2021).

To improve plant yield during drought stress, there are two pivotal indices we must consider: water use efficiency and photosynthesis. Drought always negatively affects the plants due to the lack of both water and photosynthetic products, which directly reduces plant biomass production (Proietti et al., 2008; Rouphael et al., 2008). Therefore, it is imperative to select rootstocks capable of raising the water and nutritional status of scions. (López-Marín et al., 2017). Previous studies found that some drought-resistant pepper, watermelon, and tomato rootstocks could alleviate the negative effects on roots and improve their water use efficiency to increase yields under limited irrigation conditions (Poudyal et al., 2017; Al-Harbi et al., 2018; Yavuz et al., 2020). Thus, the less the reduction of photosynthetic ability, the higher the yield in grafted vegetables (López-Marín et al., 2017; Gisbert-Mullor et al., 2020).

The fruit quality includes nutritional value, taste, shape, uniformity, and odor (Khadivi-Khub and Anjam, 2016; Ellenberger et al., 2021). Drought causes yield reduction, but its effects on fruit quality are still undetermined. Nevertheless, grafting has been widely recommended as a useful tool to help plants acclimatize to drought conditions. Many studies have found that grafting altered the composition of secondary metabolites to improve the fruit’s nutrients and flavor (Čolić et al., 2021; Ellenberger et al., 2021; Seymen et al., 2021; El-Mogy et al., 2022). By using drought-tolerant ‘Zarina’ as the rootstock, the grafted tomato plants showed a moderate accumulation of antioxidant compounds, sugars, organic acids, and minerals, which ultimately improved the quality of fruits under moderate water stressed conditions (Sánchez-Rodríguez et al., 2012). Similarly, under water stress, ‘Durinta’ cultivars that were grafted with ‘Beaufort’ rootstocks had accumulated vitamin C and total soluble solids, which could improve tomato quality (Abdulaziz et al., 2017).

Due to the different genotypes of scions and rootstocks, connecting the two may cause genetic limitations. For example, in some grafted combinations, when drought-resistant plants and drought-sensitive plants were used as rootstocks and scions, respectively, the grafted plants had better drought resistance than the contrary combinations where drought-resistant plants and drought-sensitive plants were respectively used as scions and rootstocks. However, sometimes despite the drought-resistant soybean being used as the scion or the rootstock, the grafted plants could maintain photosynthesis (Li et al., 2019; Spiral et al., 2022). This genetic limitation is more obvious when we consider fruit quality. The scion variety theoretically determines the fruit composition in grafted plants. Due to the genetic limitation in grafted plants, varied rootstocks can drastically influence and alter the agronomic traits as well, whereas the role of the scion will be weakened (Zhang et al., 2016). Sometimes when drought-resistant rootstock was grafted with high-yielding scion, this combination did not always have high yield and fruit quality, thus indicating that the effect of grafting partly depended on the grafted combination (Sánchez-Rodríguez et al., 2012). Ignoring the scion-rootstock interaction or randomly choosing only vigorous rootstock and high-yielding scion is not a good strategy to improve yield and fruit quality (Karunakaran and Ilango, 2019). Therefore, besides the drought-resistant rootstock, considering the scion-rootstock interaction, and choosing the best combination for better drought acclimatization and the consequent increase in yield and fruit quality is a commonly used strategy (Khadivi-Khub and Anjam, 2016; Al-Harbi et al., 2018). Various grafted combinations must be thoroughly evaluated through studies in the field for the final identification of the most suitable combination (Karunakaran and Ilango, 2019).

## Phytohormones

**Figure 2:**
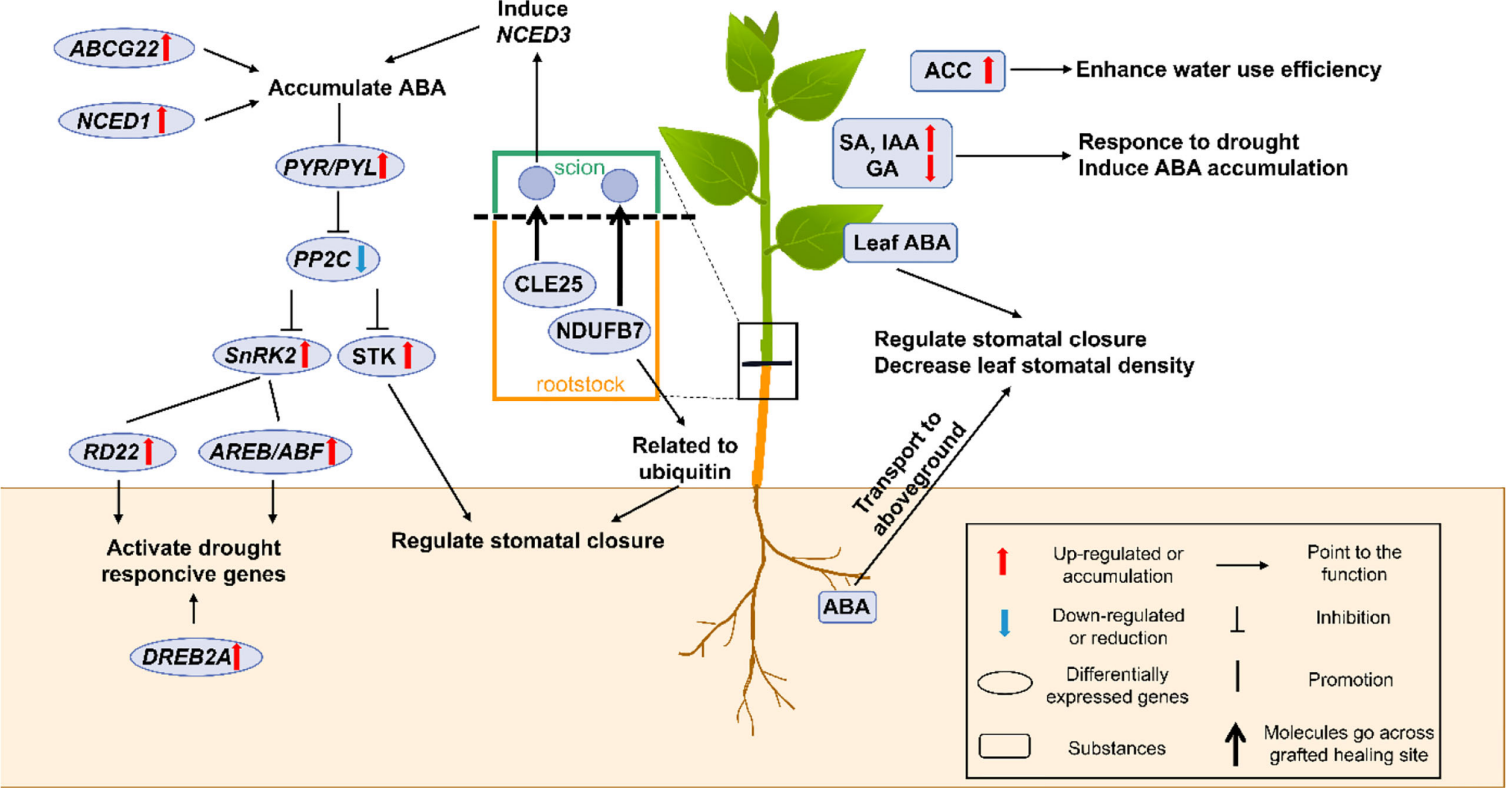
A schematic model shows the physiological and molecular mechanisms that enhancing drought resistance by the accumulation of phytohormones (especially ABA) in grafted plants. In grafted plants, the green part represents for the scion, and the brown part represents for the rootstock. The box shows the figure notes. ABA, abscisic acid; *ABCG22*, *ATP-binding cassette subfamily G transporter 22*; ACC, ethylene 1-amynocyclopropane-1-carboxylic; *AREB/ABF*, *ABA-responsive element binding protein*/*ABA-responsive element binding factor*; CLE25, clavata3/embryo-surrounding region-related 25; *DREB2A*, *dehydration-responsive element-binding protein 2 A*; GA, gibberellin; IAA, Indole-3-acetic acid; *NCED1* and *3*, *9-cis-epoxycarotenoid dioxygenase 1* and *3*; NDUFB7, NADH-ubiquinone oxidoreductase B18 subunit; PP2C, protein phosphatase 2C; *PYR/PYL*, *pyrabactin resistance1/pyr1-like*; *RD22*, *responsive to dehydration 22*; SA, salicylic acid; *SnRK2*, sucrose non-fermenting-1-related protein kinase 2; *STK*, *serine/threonine-protein kinase-transforming protein*.

Abscisic acid (ABA) is the most important phytohormone for intensifying plant drought resistance *via* various morpho-physiological and molecular processes. Other phytohormones, like jasmonic acid (JA), salicylic acid (SA), ethylene (ET), auxins (IAA), gibberellins (GAs), cytokinins (CKs), and brassinosteroids (BRs), are also important for water deficit conditions (Ullah et al., 2018). These phytohormones usually cross-talk with each other to promote the plant’s survival during drought stress. In grafted plants, as shown in **Figure 2**, roots can primarily sense soil water deficit and activate the appropriate signaling molecules to resist drought stress (Acharya and Assmann, 2009; Gaion et al., 2018; Zhang et al., 2019a).

### ABA

Stomata are vital organs that exchange gas and water with the external environment to ensure maximum CO_2_ absorption for photosynthesis and efficient control of optimal transpiration during plant life. To cope with drought stress, plants mainly use stomatal movement to control transpiration and the stomatal density changes for a long-term drought period (Marguerit et al., 2012). ABA is considered a key regulator in stomatal closure and is important in drought stress resistance. Extensive studies on the synergetic role of drought and ABA have already been conducted (Muhammad Aslam et al., 2022). They have shown that ABA triggered diverse physiological and molecular responses, like stomatal closure, cuticular wax deposition, root system modulation, activation of transcriptional and post-transcriptional gene expression, and metabolic changes in regular plants (Muhammad Aslam et al., 2022). In grafted plants, the mechanisms of ABA-mediated stomatal movement under drought stress have also been reported in detail. Under short-term drought, ABA was synthesized in roots and then transported into the guard cells to trigger stomatal closure and reduced transpiration *via* the branch signal cascade (Wilkinson and Davies, 2002; Allario et al., 2013; Sarwat and Tuteja, 2017). Under long-term drought, decreased stomatal density was found in the newly developed leaves of the grafted plants, thereby demonstrating that the high ABA content in new leaves decreased the leaf stomatal density and enabled the grafted plants to maintain long-term drought resistance (Tanaka et al., 2013; Liu et al., 2016). Despite their usual function, studies have also shown that the accumulated ABA in the grafted cucumber/luffa combination might also improve the activity of antioxidative enzymes (Liu et al., 2016). Since the ABA-dependent signaling pathway is one of the stress signal transduction-related pathways, ABA can be the signal that triggers the expression of drought-resistant genes in grafted plants. This will be discussed in the following parts (Gong et al., 2015; Du et al., 2018; Silva et al., 2018). Therefore, ABA has multiple functions in grafted plants and its significant accumulation of ABA is a helpful feature for selecting drought-resistant grafted combinations.

### Other phytohormones

SA is a common defensive hormone that also participates in drought resistance (Santana-Vieira et al., 2016). Studies revealed that foliar SA accumulation was detected in grafted citrus plants, which could positively regulate stomatal closure (Santana-Vieira et al., 2016). Furthermore, exogenous SA application before drought treatment triggered the ABA synthesis during water deficiency (Bandurska and Stroinski, 2005; Santana-Vieira et al., 2016). IAA is synthesized in the root tip or shoot apex and has been thoroughly studied to date (Ullah et al., 2018). It was reported that IAA could regulate drought resistance by adjusting root architecture and promoting the ABA-responsive genes’ expression in citrus rootstocks (Shi et al., 2014). The transgenic *gretchen hagen 3* (*GH3*) RNAi plants (*GH3* silencing) used as rootstock caused IAA accumulation, which further induced RSA enlargement to absorb water and maintain water use efficiency (Jiang et al., 2022). GA plays a vital role throughout the plant life cycle (Ullah et al., 2018). Studies have revealed that a low GA level is useful for improving plant drought resistance (Ullah et al., 2018). Similarly, the high ABA content triggered by the low GA content in the grafted tomato plants could finally enhance their drought resistance (Gaion et al., 2018). However, the role of other phytohormones, like JA, CKs, and BRs during drought treatment, is poorly studied in grafted plants. In summary, the changes in the SA, IAA, and GA contents are commonly accompanied by ABA synthesis and they interact synergistically and antagonistically to regulate each other in the grafted plants (Santana-Vieira et al., 2016; Gaion et al., 2018; Sousa et al., 2022).

Additionally, in the grafted tomato plants, the root-derived precursor of ethylene 1-aminocyclopropane-1-carboxylic (ACC) could increase the fruit yield and agronomic water use efficiency (Cantero-Navarro et al., 2016). Applying exogenous melatonin onto the rootstocks improved their drought resistance by regulating the key metabolic pathways, like the phenylpropanoid pathway, chlorophyll and carotenoid biosynthesis, carbon fixation, and sugar metabolism (Lunn et al., 2014; Sharma et al., 2020). However, how the accumulation of melatonin and ACC can enhance drought resistance in grafted plants still needs further investigation.

## Signaling pathway

### ABA-dependent pathway

As shown in **Figure 2**, the ABA-dependent signaling pathway is crucial in regulating stomatal movement and activating drought-responsive gene expression in plants (Ullah et al., 2018). This includes the accumulation and signal transduction pathways. Roots are the main sites of ABA biosynthesis, followed by leaves. 9-cis-epoxycarotenoid dioxygenase (NCED) is the key rate-limiting enzyme in ABA biosynthesis (Seo and Koshiba, 2002; Ksouri et al., 2016; Ali et al., 2020). In autotetraploid clones of citrus rootstock (Rangpur lime), *NCED1* was found highly up-regulated, and it improved ABA accumulation that ultimately enhanced drought resistance (Allario et al., 2013). Additionally, an ABA transporter gene, *ATP-binding cassette subfamily G transporter 22* (*ABCG22*) was also indirectly up-regulated in the cucumber/luffa grafted combination, thereby suggesting an ABA accumulation (Liu et al., 2016). There are molecules crossing grafted healing sites to build the scion-rootstock communication. Grafting experiments have demonstrated that clavata3/embryo-surrounding region-related 25 (CLE25) was transported from the roots to leaves, where it induced *NCED3* expression and ABA accumulation to promote stomatal closure under drought stress (Takahashi et al., 2018).

Besides the ABA accumulation, the leaf transcriptome showed that drought resistance in scions induced by rootstocks was related to transcriptional activation of ABA-dependent signaling pathway genes (Goncalves et al., 2019). ABA sensing and signaling are mediated by three classes of proteins: *PYR/PYL*, *PP2C*, and *SnRK2*. Being a hormonal signal, ABA first binds to the pyrabactin resistance1/pyr1-like (PYR/PYL) receptor, followed by constant binding with protein phosphatase 2C (PP2C) to form a ternary complex, which triggers the release of the transcription factor *sucrose non-fermenting-1-related protein kinase 2* (*SnRK2*) (Zhang et al., 2019b). This is the main ABA signal transduction pathway. The up-regulated *PYR/PYL*, and *SnRK2* along with down-regulated *PP2C* were detected in the grafted tomato plants during drought stress, thereby indicating that the ABA signal transduction pathway was activated in the grafted tomato (Zhang et al., 2019b). *SnRK2* plays important role in ABA-responsive stomatal closure and ABA-dependent gene expression. *ABA-responsive element binding protein*/*ABA-responsive element binding factor* (*AREB/ABF*) is activated through multi-site phosphorylation of the conserved domains by *SnRK2* in the grafted tobacco plants, and it directly acts on numerous drought-responsive genes (Liu et al., 2014; Ullah et al., 2018; Soma et al., 2021). Furthermore, the activated SnRK2 induces stomatal closure through downstream regulation of ion channels and transcription factors (Soma et al., 2021). For example, the serine/threonine-protein kinase (STK) transported from the pumpkin rootstocks to cucumber scions participated in ABA-dependent stomatal movement by phosphorylating the ion channels (Staples, 2003; Ali et al., 2020). Moreover, molecules like the NADH-ubiquinone oxidoreductase B18 subunit (NDUFB7), transported from *Arabidopsis* rootstock to the tobacco scion, were related to ubiquitin, which was an important drought stress response occurring through regulating ABA signals and stomatal aperture (Notaguchi et al., 2015; Yang et al., 2017; Pan et al., 2020; Yu et al., 2020). Thus, we can speculate that ubiquitination and phosphorylation exist in the ABA-dependent signaling pathway and play important roles in grafted plants (Soma et al., 2021).

Gene overexpression in scions can also affect the ABA-dependent signaling pathway and enhance their drought resistance. *YTH domain-containing RNA binding proteins* (*YTPs*) are important in conferring drought resistance to plants (Li et al., 2014; Wang et al., 2017). The overexpression of *YTP1* in transgenic apple scions promotes the expression of the *ABF3* and *stress response monitor gene responsive to dehydration 22* (*RD22*) genes, which were involved in the ABA-dependent pathway to stimulate stomatal aperture reduction and improve water use efficiency under long-term drought-stressed conditions (Guo et al., 2019). Researchers also pointed out that grafted plants with *YTP1* transgenic apple being used as the scions exhibited better drought resistance (Guo et al., 2019).

### ABA-independent pathway

Besides the ABA-dependent signaling pathway, there are ABA-independent pathways that activate the plant drought defense system (**Figure 2**). According to microarray analysis in *Arabidopsis*, there were several pathways that independently were abiotic stress-responsive and one such important pathway is the dehydration-responsive element-binding protein (DREB) regulon (Agarwal et al., 2006). The transcription factor *DREB2* is involved in the ABA-independent pathway and uses the DRE *cis*-acting element to activate the drought responsive genes (Agarwal et al., 2006). In the grafted citrus and tomato plants, the upregulated *DREB2A*, a key drought response regulator, was detected, thus indicating that the ABA-independent pathway was activated to resist drought stress (Gaion et al., 2018; Goncalves et al., 2019).

## Osmotic adjustment

**Figure 3:**
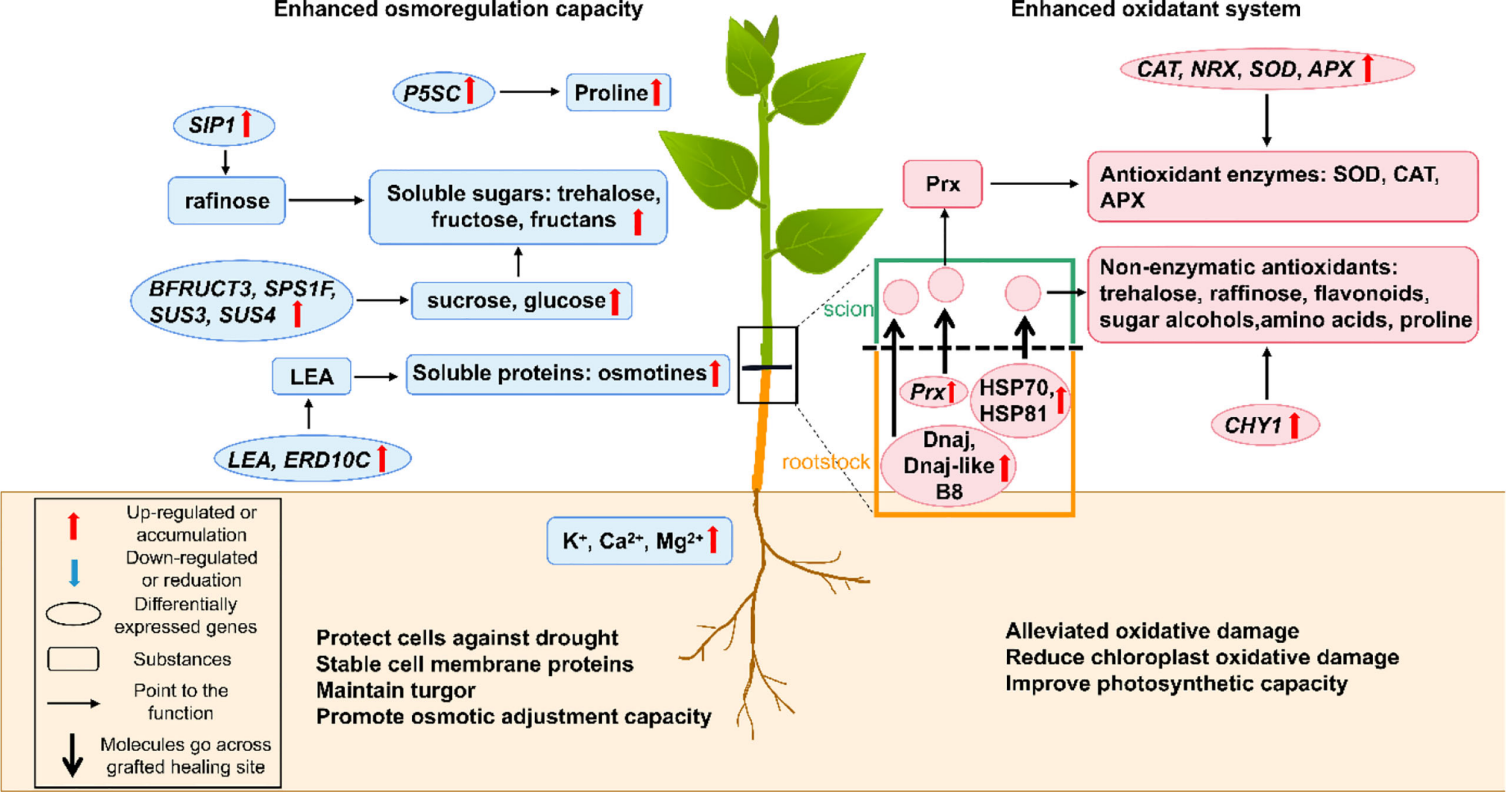
A schematic model shows the physiological and molecular mechanisms that enhancing drought resistance by osmoregulation and anti-oxidant system in grafted plants. The green part represents for the scion while the brown part represents for the rootstock. The box shows the figure notes. APX, ascorbate peroxidase; *BFRUCT3*, *β-fructosidase 3*; CAT, catalase; *CHY1*, *chloroplast β-carotene hydroxylase*; Dnaj, Chaperone protein DnaJ; Dnaj-like B8, DnaJ-like subfamily B member 8; ERD10C, early responsive to dehydration 10C; GA, gibberellin; HSP70, 70-kDa heat shock protein; HSP81, heat shock protein 81.4; *LEA*, *late embryogenesis abundant protein*; NRX, nucleoredoxin; *P5SC*, *Δ-1-pyrroline-carboxylate synthase*; *Prx*, *peroxiredoxin*; *SIP1*, *raffinose synthase*; SOD, superoxide dismutase; *SPS1F*, *sucrose phosphate synthase 1F*; *SUS3 and 4*, *sucrose synthase 3* and *4*.

Drought causes water deficiency and osmotic stress in plant cells, leading to ion imbalance and adversely affecting cellular functions (Zia et al., 2021). Under low water potential, the osmoregulation of grafted plants depends on the accumulation of different osmotically active compounds, which can improve their water retention ability and water use efficiency (**Figure 3**) (Ozturk et al., 2021). Due to the higher osmoregulatory capacity, grafted plants with drought-resistant grapevine rootstocks showed greater drought tolerance than those with drought-sensitive grapevine rootstocks (Lucini et al., 2020). Thus, choosing the right rootstocks is pivotal for grafted plants.

Proline is one of the most important osmoprotectants that can stabilize membrane and protein conformation by forming protective films with water molecules on their surface (Ozturk et al., 2021). In many grafted plants, proline was found accumulating in plant tissues to improve drought resistance (Liang et al., 2013; Penella et al., 2014; Ozturk et al., 2021). The proline accumulation in plant tissues under drought conditions can be obtained from the activation of proline biosynthesis, inactivation of proline degradation, protein hydrolysis, or oxidative inhibition of protein synthesis (Szabados and Savoure, 2010; Ozturk et al., 2021). In higher plants, the glutamate and the ornithine pathways of proline biosynthesis are known, with the former being the primary pathway in response to osmotic stress (Ozturk et al., 2021). *Δ-1-pyrroline-carboxylate synthase* (*P5SC*) encoded a key enzyme involved in proline anabolism that converts L-glutamate to glutamate γ-semialdehyde and was found to be up-regulated in the grafted plum plants. Thus, the glutamate pathway of proline biosynthesis was proven to be activated in grafted plants under water-deficit conditions (Jimenez et al., 2013). Additionally, when suffering from repeated-drought stress, the grafted plants continuously accumulated osmoregulatory substances by changing epigenetic modifications. For example, during the repeated-drought conditions, turnip rootstock altered the epigenetic modification of the *Δ1-pyrroline-5-carboxylate synthetase 1-2* (*P5CS1-2*) gene and improved its expression, which led to proline accumulation, when compared with the none-grafted rapeseed (Luo et al., 2020). However, under recurring drought stressed conditions, how the rootstock signal mediates the histone H3K4me3 modification of the P5CS1-2 locus in the scion to regulate proline biosynthesis, is still not understood clearly.

The higher contents of soluble sugars and soluble proteins were found in the grafted poplar, tomato, and citrus plants under drought stress, which generated a stronger osmotic adjustment effect (Han et al., 2018; Zhang et al., 2020; Dong et al., 2021). Soluble proteins, *e.g*., late embryogenesis abundant (LEA) proteins and osmotins, are common osmoprotectants that stabilize cell membrane proteins and promote osmoregulation capacity (Ozturk et al., 2021). Many genes involved in soluble protein biosynthesis were found up-regulated in drought-resistant grafted plants. The *LEA5* and *early responsive to dehydration 10C* (*ERD10C*) genes encoding dehydrins in the grafted tobacco plants and genes encoding osmotins in drought-resistant rootstocks were up-regulated during drought treatment (Kovacs et al., 2008; Witte et al., 2010; Liu et al., 2014; Yildirim et al., 2018; Yang et al., 2021). Soluble sugars, *e.g*., sucrose, trehalose, fructose, and fructan, can significantly reduce the cell osmotic potential, stabilize cellular membrane and protein conformation, or promote the osmotic adjustment and turgor maintenance as osmotic agents (Goncalves et al., 2019; Ozturk et al., 2021). Soluble sugars biosynthesis genes were also up-regulated in drought-resistant grafted plants under drought conditions. The *raffinose synthase* (*SIP1*) gene was found up-regulated in the grafted peach plants (Jimenez et al., 2013). Furthermore, the genes encoding starch branching enzymes increased the starch content in the cucumber/pumpkin grafted combination (Davoudi et al., 2022). The carbohydrate metabolism-related genes *β-fructosidase 3* (*BFRUCT3*), *sucrose phosphate synthase 1F* (*SPS1F*), *sucrose synthase 3* and *4* (*SUS3* and *SUS4*) were upregulated in grafted drought-resistant rootstocks (Goncalves et al., 2019).

The concentrations of inorganic ions also affect osmotic regulation, as they are closely related to ion pumps (Yang et al., 2021). For example, the K^+^ pump can change the cell osmotic potential by regulating the inorganic ion concentration both inside and outside the cells (Yang et al., 2021). Thus, the accumulated inorganic ions (K^+^, Ca^2+^, and Mg^2+^) in grafted plants protect cell membrane integrity and enhance osmoregulatory capacity (Zhang et al., 2020). Additionally, the osmotic substances, like glycine betaine, sugar alcohols, and polyamines, are also drought-inducible. However, this phenomenon is poorly reported in grafted plants (Ozturk et al., 2021).

## Antioxidative regulation

Drought leads to excessive ROS accumulation in plant chloroplast, mitochondria, and peroxisomes, thereby resulting in membrane peroxidation, enzyme inactivation, protein degradation, and even cell death (Dubey et al., 2021; Qamer et al., 2021). The grafted plants could improve drought resistance by activating the antioxidative defense system to scavenge the excess stress-induced ROS being generated (**Figure 3**) (Sanchez-Rodriguez et al., 2012).

### Antioxidative enzymes

Oxidative damage is first alleviated by antioxidant enzymes including superoxide dismutase (SOD), catalase (CAT), ascorbate peroxidase (APX), and peroxidase (POD). It is a passive mechanism primarily mediated by ROS overproduction (Sucu et al., 2017; Dubey et al., 2021). Besides ROS, ABA and free polyamines could also activate the antioxidant enzymes in grafted plants during drought stress (Liu et al., 2016; Sanchez-Rodriguez et al., 2016). SOD is a metalloenzyme that catalyzes superoxide anion (O^2-^) molecules into oxygen (O_2_) and hydrogen peroxide (H_2_O_2_) (Laxa et al., 2019). CAT, APX, and POD can convert H_2_O_2_ into water (H_2_O) and O_2_ (Laxa et al., 2019). High activity and content of these enzymes alleviate oxidative stress by reducing ROS production in the scions of grafted plants with drought-resistant poplar, citrus, apple, cucumber, and tobacco rootstocks (Liu et al., 2012; Liu et al., 2014; Melnikova et al., 2017; Chen et al., 2020; Balfagon et al., 2021; Shehata et al., 2022). Furthermore, the high antioxidant enzymatic activity of these grafted plants can reduce chlorophyll decomposition, alleviate chloroplast membrane damage, and improve photosynthesis (Sanchez-Rodriguez et al., 2012; Zhang et al., 2019a). Several up-regulated genes related to antioxidative enzyme biosynthesis were detected in grafted plants having drought-resistant rootstocks. The *SOD* and *APX* genes were up-regulated in citrus grafted plants (Goncalves et al., 2019). The *nucleoredoxin* (*NRX*) encodes a protein that protects the CAT enzyme from abiotic stress-induced damage (Kneeshaw et al., 2017). The up-regulated *NRX* and *CAT* genes could strengthen the antioxidant system in the grafted cucumber/pumpkin combination (Davoudi et al., 2022). Additionally, the mobile mRNA, encoding peroxiredoxin (Prx) (an antioxidant cysteine-dependent peroxidase) was also identified in the grafted plants (Davoudi et al., 2022). We believe that drought stress-induced antioxidant system activation is a trait transmissible from the rootstock to the scion, which depends on scion-rootstock communication (Balfagon et al., 2021). Additionally, previous studies showed that drought-resistant plants induced greater activation of the ascorbate-dependent scavenging system. Contrastingly, the drought-sensitive plants only activated the glutathione-dependent scavenging system, with only moderate induction or even down-regulation of the ascorbate-dependent system (Laxa et al., 2019). When they used as rootstocks grafted onto the same scions, this different antioxidative activated mechanisms existed between two grafted combinations or not can be explored further in depth.

### Non-enzymatic antioxidants

Drought resistance can also be improved by non-enzymatic antioxidants. Flavonoids, the common non-enzymatic antioxidants, could enhance antioxidative capacity (Lopez-Hinojosa et al., 2021). Studies have shown that most flavonoid biosynthesis-related genes were up-regulated in grafted plants (Lopez-Hinojosa et al., 2021). Furthermore, it has been reported that trehalose and raffinose in the citrus rootstock, and sugar alcohols (*e.g.*, sorbitol) and amino acids (*e.g.*, tyrosine, aspartic acid, methionine, ornithine, and tryptophan) in drought-resistant rootstocks could support the antioxidant system and scavenge ROS to protect the membrane integrity (Jimenez et al., 2013; Keunen et al., 2013; Lunn et al., 2014; Santana-Vieira et al., 2016; Sousa et al., 2022). It also indicated that the proline accumulation in the plants grafted with drought-resistant rootstocks could stabilize the membrane and proteins under water-deficient conditions (Jimenez et al., 2013; Schneider et al., 2018; Ozturk et al., 2021). Some photosynthesis-related molecules may play antioxidant roles in grafted plants. Decreased tetrapyrrole, the chlorophyll intermediates in grafted grapevine plants, could scavenge the excess ROS (Lucini et al., 2020). Carotenoid is also another well-known non-enzymatic antioxidant (Laxa et al., 2019). Upregulation of the *chloroplast β-carotene hydroxylase* (*CHY1*), encoding a carotenoid biosynthesis enzyme, indicated that the accumulated carotenoid could enhance osmoregulation in grafted citrus plants (Goncalves et al., 2019). DnaJ-like subfamily B member 8 (DnaJ-like B8) and Chaperone protein DnaJ (DnaJ), which was transported from the pumpkin rootstock to cucumber scion, helped prevent protein misfolding and aggregation, thus contributing to stress tolerance, redox maintenance and photosynthetic balance (Wang et al., 2014; Amiya and Shapira, 2021; Davoudi et al., 2022). The 70-kDa heat shock protein (HSP70) and heat shock protein 81.4 (HSP81) were transported from the pumpkin to the cucumber and they accumulated in the cucumber scions during drought stress to enhance membrane stability and detoxify the accumulating ROS (Ul Haq et al., 2019; Davoudi et al., 2022). Additionally, ascorbate (ASA), glutathione (GSH) and α-tocopherol are important plant non-enzymatic antioxidants, which need further exploration in grafted plants (Schneider et al., 2018; Hasanuzzaman et al., 2019).

## Summary and outlook

The selection of suitable rootstocks or scions is crucial in generating the drought resistance of grafted plants. Besides enabling the grafted plants to regulate general morphological, physiological, and molecular processes and allow adaptive changes to improve drought resistance, the graft-responsive genes, especially through the exchange of genetic information between rootstocks and scions, are important in improving plant performance under drought stress. It also is vital in promoting water use efficiency, osmoregulation, anti-oxidant-mediated stress tolerance, etc. Furthermore, future research can focus more on the improvement of drought resistance of grafted trees, which is a potential research avenue that can be applied in arid and semi-arid areas. Moreover, with the increasing in-depth research, we believe that the scion-rootstock communication is a complex bidirectional feedback loop that is crucial for enhancing drought resistance *via* transport molecules. Therefore, studying the scion-rootstock communication holds the key to future understanding and improving plant drought resistance.

## Author contributions

LY collected and analyzed the data, and written the manuscript. LX and YZ collected some data. QH and SZ designed the framework, given some advises and revised the manuscript. All authors read and approved the final manuscript.

## Funding

This work was supported by the National Natural Science Foundation of China (32101478), the Second Tibetan Plateau Scientific Expedition and Research Program (2019QZKK0404) and China Postdoctoral Science Foundation (2021M702360).

## Acknowledgments

The authors would like to express their gratitude to EditSprings (https://www.editsprings.cn) for the expert linguistic services provided.

## Conflict of interest

The authors declare that the research was conducted in the absence of any commercial or financial relationships that could be construed as a potential conflict of interest.

## Publisher’s note

All claims expressed in this article are solely those of the authors and do not necessarily represent those of their affiliated organizations, or those of the publisher, the editors and the reviewers. Any product that may be evaluated in this article, or claim that may be made by its manufacturer, is not guaranteed or endorsed by the publisher.

## Glossary

**Table:**

ABA	abscisic acid
*ABCG22* ACC	ATP-binding cassette subfamily G transporter 22ethylene 1-aminocyclopropane-1-carboxylic
*AREB/ABF*	*ABA-responsive element binding protein/ABA-responsive element binding factor*
ASA	Ascorbate
APX	ascorbate peroxidase
*BFRUCT3*	*β-fructosidase 3*
BRs	brassinosteroids
*Cab*	*Chlorophyll a/b binding protein*
CAT	Catalase
*CHY1*	*Chloroplast β-carotene hydroxylase*
CKs	Cytokinins
CLE25	Clavata3/embryo-surrounding region-related 25
*COMT*	*Caffeic acid 3-O-methyltransferase*
DEGs	Differentially expressed genes
*Dnaj*	*Chaperone protein DnaJ*
*Dnaj-like B8*	*DnaJ-like subfamily B member 8*
*DREB2A*	*Dehydration-responsive element-binding protein 2 A*
*DRM*	*Drought-responsive mobile gene*
*ERD10C*	*Early responsive to dehydration 10C*
ET	Ethylene
*FADs*	*Fatty acid desaturase enzymes*
GA	Gibberellin
*GH3*	*Gretchen hagen 3*
*GNS1*	*Glucan endo-1,3-β-glucosidase*
GPX	glutathione peroxidase
GSH	Glutathione
*HMGB*	*High mobility group B protein 2*
H_2_O	water
H_2_O_2_	hydrogen peroxide
*HSP81*	*Heat shock protein 81.4*
*HSP70*	*70-kDa heat shock protein*
IAA	Indole-3-acetic acid
JA	jasmonic acid
*KCS*	*3-ketoacyl-CoA synthase*
*LEA5*	*Late embryogenesis abundant protein 5*
*LhcI*	*Photosystem I light-harvesting chlorophyll a/b-binding protein*
*NCED*	*9-cis-epoxycarotenoid dioxygenase*
*NDUFB7*	*NADH-ubiquinone oxidoreductase B18 subunit*
*NRT1/PTR*	*Nitrate transporter 1/peptide transporter*
*NRX*	*Nucleoredoxin*
O_2_	oxygen
O^2−^	superoxide anion
*PIP*	*Plasma membrane intrinsic proteins*
*P5SC*	*Δ-1-pyrroline-carboxylate synthase*
POD	Peroxidase
*PP2C*	*Protein phosphatase 2C*
PR	Pathogenesis-related
*psaA*	*Photosystem I P700 chlorophyll a apoprotein A1*
*psaB*	*Photosystem I P700 chlorophyll a apoprotein A2*
*psbB*	*Photosystem II CP47 reaction center protein*
*psbD*	*Photosystem II protein D2*
*Prx*	*Peroxiredoxin*
*PYR/PYL*	*Pyrabactin resistance1/pyr1-like*
*RbcL*	*Ribulose-1,5-diphosphate carboxylase/oxygenase large subunit*
*RbcS*	*Ribulose-1,5-bisphosphate carboxylase/oxygenase small subunit*
*RD22*	*Responsive to dehydration 22*
ROS	Reactive oxygen species
RSA	Root system architecture
Rubisco	Ribulose-1,5-diphosphate carboxylase/oxygenase
SA	Salicylic acid
*SIP1*	*Raffinose synthase*
*SnRK2*	*Sucrose non-fermenting-1-related protein kinase 2*
*SPS1F*	*Sucrose phosphate synthase 1F*
SOD	Superoxide dismutase
STK	Serine/threonine-protein kinase-transforming protein
*SUS3 and 4*	*Sucrose synthase 3 and 4*
*SWEET14*	*Bidirectional sugar transporter*
*TIP*	*Tonoplast intrinsic protein*
*YTPs*	*YTH domain-containing RNA binding proteins*
