# Supplementary material for: Grafting enhances plants drought resistance: Current understanding, mechanisms, and future perspectives
Source: Front Plant Sci. 2022 Oct 6;13:1015317. doi: 10.3389/fpls.2022.1015317 (PMC9583147; doi:10.3389/fpls.2022.1015317)
Supplement: Supplementary file 1 [file Table_1.docx]

**Supplementary Table 1.** The yield and quality of grafted plants during drought.

| Type | Yield | Quality | Scion/Rootstock | | Reference |
| --- | --- | --- | --- | --- | --- |
| Vegetables | Increased | Improved by high levels of sugars and organic acids, sweetness index and sugars: acids ratio, as well as important minerals, such as K and Mg | *Solanum lycopersicum* Mill (Josefina) / *S. lycopersicum* Mill (Zarina) | Sánchez-Rodríguez et al., 2012 | |
|  | Increased | Improved by the accumulation of total soluble solid contents, titratable acidity and vitamin C | *Cucumis sativus* L./ *Cucurbita maxima* × *C. moschata* | Al-Harbi et al., 2018 | |
|  | Increased | No mentioned | *S. lycopersicum* Mill / *S. habrochaites* (ILs) | Poudyal et al., 2017 | |
|  | Increased | Improved | *Capsicum annuum* L. ‘Maestral’ F1 / *C. annuum* L. F1 NIBER^®^ | Gisbert-Mullor et al., 2020 | |
|  | Increased, including fruit number and weight | No mentioned | *C. annuum* L. ‘Herminio’ F1 / commercial rootstock Creonte | López-Marín et al., 2017 | |
|  | Increased | Improved | *C.annuum* L. ‘Herminio’ F1 / commercial rootstock Terrano |  |  |
| Fruits | Increased by improvement in nutritional status, high photosynthesis and water uptake | Improved by titratable acidity, K, and Mg concentrations improvement | *Citrullus lanatus* (Thunb.) / *Cucurbita moschata* Duchesne·×  *C. maxima* Duchesne | Rouphae et al. 2008 | |
|  | Increased | Improved |  | Proietti et al., 2008 | |
|  | No mentioned | Improved by high levels of sugars | Crimson Tide *Citrullus lanatus* (Thunb.) cultivar / *Cucurbita moschata* Duchesne·×  *C. maxima* Duchesne | Seymen et al., 2021 | |
| Trees | Increased, including kernel quality | Improved by high contents of oleic acid, flavanols and acidic subunits of amandin | *Prunus dulcis* ‘Texas’ / peach × almond hybrid ‘GF 677’ | Čolić et al., 2021 | |

Note: The last two rows demonstrate which the grafted combinations and references the molecules are from.
